# Tick-Borne Encephalitis Virus and *Borrelia burgdorferi* Seroprevalence in Balkan Tick-Infested Individuals: A Two-Centre Study

**DOI:** 10.3390/pathogens12070922

**Published:** 2023-07-09

**Authors:** Dejan Jakimovski, Sofija Mateska, Emilija Dimitrova, Mile Bosilkovski, Dragana Mijatović, Verica Simin, Ivana Bogdan, Jasmina Grujić, Zorana Budakov-Obradović, Eleftherios Meletis, Polychronis Kostoulas, Alejandro Cabezas-Cruz, Pavle Banović

**Affiliations:** 1 Faculty of Medicine, Ss. Cyril and Methodius University in Skopje, 1000 Skopje, North Macedonia; milebos@yahoo.com; 2 University Clinic for Infectious Diseases and Febrile Conditions, 1000 Skopje, North Macedonia; mateskasofija@hotmail.com; 3 Department of Infectious Diseases, City General Hospital 8th September, 1000 Skopje, North Macedonia; emilija.dimitrova@yahoo.com; 4 Department for Research and Monitoring of Rabies and Other Zoonoses, Pasteur Institute Novi Sad, 21000 Novi Sad, Serbia; draganav77@gmail.com; 5 Department of Microbiology, Pasteur Institute Novi Sad, 21000 Novi Sad, Serbia; vericasimin071@gmail.com (V.S.); ivana.basaric@gmail.com (I.B.); 6 Blood Transfusion Institute Vojvodina, 21000 Novi Sad, Serbia; jasmina.grujic@mf.uns.ac.rs (J.G.); zorana.budakov-obradovic@mf.uns.ac.rs (Z.B.-O.); 7 Faculty of Medicine in Novi Sad, University of Novi Sad, 21000 Novi Sad, Serbia; 8 Faculty of Public and One Health, School of Health Sciences, University of Thessaly, 24410 Karditsa, Greece; emeletis@outlook.com (E.M.); pkost@uth.gr (P.K.); 9 Anses, INRAE, Ecole Nationale Vétérinaire d’Alfort, UMR BIPAR, Laboratoire de Santé Animale, F-94700 Maisons-Alfort, France; alejandro.cabezas@vet-alfort.fr; 10 Clinic for Lyme Borreliosis and Other Tick-Borne Diseases, Pasteur Institute Novi Sad, 21000 Novi Sad, Serbia; 11 Department of Microbiology with Parasitology and Immunology, Faculty of Medicine in Novi Sad, University of Novi Sad, 21000 Novi Sad, Serbia

**Keywords:** TBEV, Lyme borreliosis, seroprevalence

## Abstract

Lyme borreliosis (LB) and tick-borne encephalitis (TBE) are important tick-borne diseases in Europe. This study aimed to investigate the seroreactivity against *Borrelia burgdorferi* and TBE virus (TBEV) in tick-infested individuals in North Macedonia and Serbia. Serum samples were collected from tick-infested individuals and from healthy individuals in the same regions. Samples were tested for anti-*Borrelia* IgG reactivity and TBEV-neutralizing antibodies. Results showed higher seroreactivity against *Borrelia* antigens in patients and healthy donors from Novi Sad compared to those from the Skopje region. However, there was no statistically significant difference between tick-infested patients and healthy donors within each region. No TBEV-neutralizing antibodies were detected in participants from Novi Sad or in the control groups, except for one person from North Macedonia who had a moderate TBEV-neutralizing reaction. The study highlights the need for improved surveillance and diagnostic capabilities for LB and TBE in these regions. It also suggests the potential existence of TBEV foci in North Macedonia. The findings provide a complementary understanding of the LB and TBE epidemiology in the studied regions; however, further research is needed to investigate the presence and distribution of *Borrelia* spp. and TBEV in ticks to assess the significance of detected seroreactivity.

## 1. Introduction

Lyme borreliosis (LB), caused by pathogenic members of *Borrelia burgdorferi* sensu lato (s.l.) complex, is the most frequent tick-borne disease in humans and is prevalent in the northern hemisphere [1]. The global incidence of LB is increasing due to various factors such as climate change, travel, occupational exposure to tick bites, and increased outdoor activities [2,3,4,5,6].

The accurate estimation of LB incidence in North Macedonia and Serbia is challenging as there is no established surveillance case definition, resulting in sporadic reporting of cases [7]. In North Macedonia, the existence of LB remains controversial due to limited diagnostic capabilities, low awareness among medical practitioners, and the lack of definitive identification of *Borrelia* spp. infecting humans. Conversely, Serbia is considered an endemic region for LB based on scientific publications assessing LB incidence and exposure in humans and tick-infested animals [8,9,10,11].

Tick-borne encephalitis virus (TBEV) is the most important viral pathogen transmitted by hard ticks. This virus belongs to the *Flavivirus* genus, Flaviviridae family. TBEV infection is often asymptomatic but can cause tick-borne encephalitis (TBE) with central nervous system (CNS) involvement in persons with full clinical manifestation of disease [12]. TBEV is classified into different genotypes [13], including European (TBEV-Eu), Siberian (TBEV-Sib), and Far-Eastern (TBEV-FE), with the latter being the most lethal and endemic in Ukraine, Latvia, Estonia, and eastern regions of Russia [14].

Within Europe, TBEV is endemic in the Central, Baltic, and East regions, forming a “TBE belt”. The belt of TBE endemicity further spans over Asia, to the far east of Russia [15]. The incidence of TBE differs due to socioeconomic, ecological, and climate-related factors, as well as immunization strategies [16,17]. Additionally, the incidence rate is strongly dependent on national diagnostic guidelines and diagnostic resources [18]. TBEV foci can appear and disappear in different regions over time [19]. Despite the presence of TBEV-infected ticks in various locations in Serbia [20], no TBEV-positive ticks have been reported in North Macedonia to date.

TBE is probably highly neglected in North Macedonia and Serbia, as a consequence of it not being considered in the differential diagnosis of CNS infections. Limited diagnostic resources further complicate the diagnosis of possible autochthonic and imported TBE cases [21]. Previous TBE case definitions used in Serbia were not aligned with European recommendations [22,23], potentially leading to misdiagnosis prior to the implementation of the TBEV neutralization assay during 2022.

This study aims to investigate the seroreactivity against *B. burgdorferi* and TBEV in tick-infested individuals in North Macedonia and Serbia, two neighbouring countries on the Balkan peninsula.

## 2. Materials and Methods

### 2.1. Study Design and Participant Enrolment

A retrospective study was conducted among individuals from North Macedonia and Serbia who sought consultation for tick infestation at the Infectious Diseases Clinic in Skopje (IDC Skopje) and the Pasteur Institute in Novi Sad (PI Novi Sad), respectively. Four weeks after their tick infestation, patients were invited to participate in the study. Those who agreed were asked to visit the IDC Skopje and PI Novi Sad for blood withdrawal. To establish control groups for each study arm, serum samples were collected from healthy individuals residing in the same regions (i.e., Skopje and Novi Sad). For all enrolled individuals, records of previous immunizations and medical history related to TBE and/or LB manifestations were examined. Additionally, demographic information such as gender and age was registered for each patient.

### 2.2. Detection of Anti-Borrelia spp. IgG

Blood samples (3 mL) were collected for each patient by phlebotomy, using BD Vacutainer^®^ SST™ Tubes (BD, Franklin Lakes, NJ, USA). The tubes were left at room temperature to allow the blood to clot, and serum was separated by centrifugation at 2000× *g* for 10 min. The separated serum samples were then inactivated at 56 °C and used for the detection of anti-Borrelia spp. IgG reactivity, using a commercial ELISA kit (recomWell Borrelia IgG, Mikrogen Diagnostik GmbH, Neuried, Germany; Cat. No. 4204). The ELISA kit included OspC and the VlsE antigens derived from *B. burgdorferi sensu stricto* (s.s.), *B. garinii* and *B. afzelii*. The assay was performed following the manufacturer’s instructions, and positive, negative, and cut-off controls provided with the kit were included. The ELISA results were interpreted qualitatively (positive or negative). A finding was considered negative if the value was <24 units/mL, and positive if the value was >24 units/mL. The number of units/mL was calculated according to the manufacturer’s instructions after measuring the optical density (O.D.) at 450 nm using an ELX800 ELISA reader (BioTek, Winooski, VT, USA) provided by Dr Miladin Kostović, Biotehnika IVD, Ratina, Serbia.

### 2.3. Detection of TBEV-Neutralizing Antibodies

The TBEV strain Neudörfl (National Collection of Pathogenic Viruses, United Kingdom; Cat. No 0201139v) was cultured in a monolayer of BHK-21/C13 cells (BS CL 8, Istituto Zooprofilattico Sperimentale Brescia, Italy) in a BSL2+ Laboratory for Vector Borne Pathogens in Pasteur Institute Novi Sad. Virus stocks were prepared at a concentration of 100 Tissue Culture Infectious Dose (TCID)/100 μL and stored at −80 °C until further use.

The micro-neutralization test (micro-NT) was conducted using a 96-well cell culture plate (Thermo Scientific™, MA, USA, Cat. No 130338). Serum samples were first inactivated at 56 °C for 30 min and tested in duplicate, with serial dilutions ranging from 1:5 to 1:40 in Glasgow Minimal Essential Medium (Biowest, France; Cat. No P0120).

In each test run, positive and negative controls, a cell control, and a virus back-titration were included. A total of 100 TCID of virus stock was added to the respective serum dilutions and incubated for one hour at 37 °C. After incubation, the serum-virus mixture was transferred to wells containing BHK21/C13 cells that were previously seeded at a concentration 2 × 10^5^ cells and incubated for five days at 37 °C in atmosphere with 5% CO_2_. The cytopathic effect (CPE) was observed in both wells for each sample. The dilution of the sample resulting in virus neutralization in 50% of the replicates (NT50) was calculated using the Spearman and Karber method [24]. A serum sample with a NT50 titre of ≥1:10 in the neutralization assay was considered a positive result.

### 2.4. Statistical Analysis

To identify specific risk groups for exposure to *B. burgdorferi* s.l. and TBEV, we conducted an analysis of clinical findings and demographic data, including gender, age, and residency. The age groups were categorized as follows: children (1–10 years), teenagers and adults (11–64 years), and seniors (≥65 years). The association between seropositivity and risk factors was tested using the relative risk (RR) measurement. To account for small case numbers and avoid overestimation of statistical significance, a Chi-square (χ^2^) association test with Yates’s correction was applied. In cases where any of the comparison groups had less than five cases, the Fisher exact probability test was utilized to create a contingency table. Statistical analysis was performed using GraphPad software v.9 (GraphPad Software Inc., La Jolla, CA, USA). Statistically significant differences were considered when *p* < 0.05.

## 3. Results

### 3.1. Patient Enrolment and Clinical Outcomes

During the year 2022, a total of 386 and 433 patients were reported to IDC Skopje and PI Novi Sad, respectively, due to tick infestation. Serum samples were obtained from 45 (11.65%, 45/386) patients in Skopje and 51 (11.77%, 51/433) patients in Novi Sad after a four-week follow-up period. None of the enrolled patients had a history of previous infections or symptoms associated with the CNS, nor had they received immunizations against TBEV or yellow fever. In addition, none of the patients developed clinical signs of LB or TBE during follow-up period. Table 1 illustrates the distribution of patients and healthy donors included in the study. Control groups for both regions consisted of healthy donors from Novi Sad (n = 62) and Skopje (n = 46).

### 3.2. Seroprevalence of Anti-Borrelia IgG and TBEV-Neutralizing Antibodies in Tick-Infested Patients and Healthy Donors from Novi Sad and Skopje Regions

**Figure 1:**
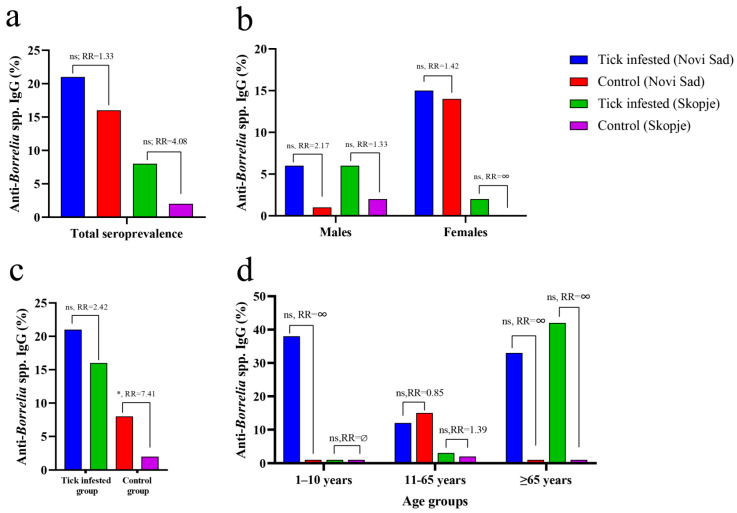
Seroprevalence of anti-Borrelia IgG in tick-infested patients and healthy donors according to gender, settlement, and age. (**a**) Comparison of total prevalence values between the studied groups. (**b**) Comparison between the groups of males and also between females from tick-infested patients and healthy donors living in Skopje and Novi Sad. (**c**) The tick-infested patients and donors were compared according to location of residence (Skopje vs. Novi Sad). (**d**) Comparisons were performed in each age group as well, despite no children or senior individuals being present in the cohort of healthy donors from Novi Sad and Skopje. RR: relative risk, indicates the probability of an individual in the groups of “tick-infested patients” to be seropositive to Borrelia. RR = ∞ (undefined) when the prevalence value equals 0 in a group. The significance of the association was also tested using Fisher’s exact test (* *p* < 0.05; ns, non-significant).

Comparisons were made between tick-infested patients and healthy donors based on overall seroprevalence (Figure 1a), gender (Figure 1b), settlement (Figure 1c), and age group (Figure 1d).

Regarding geographical regions, seroreactivity against specific Borrelia proteins (OspC and VlsE) was higher in patients and healthy donors from Novi Sad (11/51; 21.56% and 10/62; 16.12%, respectively) compared to those living in the Skopje region (4/45; 8.88% and 1/46; 2.17%, respectively) (Figure 1a). No statistically significant difference was found in anti-Borrelia IgG seroprevalence between patients and healthy donors from Novi Sad or the Skopje region (Fisher’s exact test, *p* > 0.05). Residents of the Skopje region had a four-fold higher relative risk (RR) of being seropositive against Borrelia antigens after tick infestation, while the likelihood of being seropositive after tick infestation was much lover in the Novi Sad cohort compared to the control group (Figure 1a).

There was no statistical difference in seroprevalence against Borrelia antigens between males and females from Novi Sad and the Skopje region (Fisher’s exact test, *p* > 0.05) (Figure 1b). Risk analysis showed that males from Novi Sad had twice the likelihood of being seropositive to Borrelia antigens after a tick bite, while the risk in tick-exposed males from Skopje was lower (RR = 2.17 vs. RR = 1.33, respectively). Females from Novi Sad showed a lower risk of being seropositive after a tick bite compared to males from Novi Sad (RR = 1.42 vs. RR = 2.17) but a similar risk to males from the Skopje region (RR = 1.42 vs. R = 1.33). Risk analysis for females from the Skopje region was not conducted due to the absence of seroreactivity against Borrelia antigens in the female control group (Figure 1b, Table 2).

No statistically significant difference was found in anti-Borrelia IgG seroprevalence between tick-exposed individuals from Novi Sad and Skopje (Fisher’s exact test, *p* > 0.05) (Figure 1c, Table 2). Risk analysis showed that patients from Novi Sad had a higher likelihood of being seropositive after tick infestation compared to the Skopje cohort. However, a statistically significant difference in seroprevalence was found when comparing the control groups from Novi Sad and Skopje (Fisher’s exact test, *p* = 0.022), with healthy individuals from Novi Sad having more than seven times higher chances of being seropositive to Borrelia antigens (Figure 1c). None of the analysed age groups showed a significant difference in anti-Borrelia IgG seroprevalence compared to the others. Notably, the control group of teenagers and adults from Novi Sad had a higher seroprevalence compared to the tick-exposed group of the same age, while tick-infected seniors from Skopje had a higher seroprevalence compared to same age group from Novi Sad. Due to the specific distribution of age groups in all cohorts (a low number of children’s serum samples from Novi Sad and an absence of children’s serum samples from Skopje), a comparison within the children’s age group was not possible (Figure 1d, Table 2).

TBEV-neutralizing antibodies (NT50 = 40) were found in one sample (1/45; 2.22%) from the tick-exposed group from Skopje (a 39-year-old female), while no TBEV-neutralizing antibodies were detected in tick-exposed patients and the control group from Novi Sad, nor in the control group from the Skopje region.

## 4. Discussion

LB and TBE pose significant threats to public health in Europe [25,26]. Immunization against TBE is recommended in many European countries, while in Austria and South Germany it is mandatory for all residents. However, in countries bordering the “TBE belt’’ such as Serbia, Bosnia and Herzegovina, Montenegro, and North Macedonia, the disease is likely neglected. This is due to the absence of clinical case definitions and adequate diagnostic laboratory support, leading to TBE cases being discharged as viral encephalitis of unknown etiology [18,21].

In our study, we did not find TBEV-neutralizing antibodies in participants from northern Serbia, regardless of their recent exposure to ticks. However, moderate TBEV neutralizing reaction was registered in the serum from one person from North Macedonia previously infested by a tick. Although the climate of North Macedonia is dominantly sub-Mediterranean, the existence of many mountainous areas may facilitate emergence of a stable TBEV-supporting ecosystem [19]. There is a chance that this person was exposed to the virus when entering TBEV foci within North Macedonia, or during a short-term stay in Austria 8 years ago.

Although previous studies reported TBEV seroreactivity in tick-infested individuals from northern Serbia [27] and in persons who recovered from viral meningitis and encephalitis [28], our findings did not provide evidence of TBEV exposure in Serbian patients exposed to tick bites in 2022 or in healthy control individuals from the same region. This discrepancy may be attributed to the small sample size, survivorship bias, territory sampling bias, or the absence of active TBEV foci in proximity to Novi Sad. The positive finding in only one participant from North Macedonia should be interpreted with caution. Regardless of the fact that the neutralization assay is a gold standard for confirmation of exposure to TBEV, the sensitivity and specificity of all diagnostic assays are known to be affected by disease prevalence in the examined population [29,30]. Nonetheless, residents of Serbia and North Macedonia should be considered at risk for TBEV contact, particularly when travelling to TBE-endemic countries, as there are currently no registered vaccines against TBE in these countries [21]. In given settings, TBEV-related etiology should be suspected in all cases of viral meningitis and/or encephalitis of unknown origin with or w/o myelitis in patients hospitalized in Serbia and North Macedonia, especially during the periods of spring and summer, when the incidence of TBE cases reaches a peak in Europe [31,32].

In contrast to TBEV-neutralizing antibodies, we observed a high prevalence of IgG reactive to *Borrelia* antigens in both the tick-exposed and control groups from the Novi Sad area. The seroprevalence was significantly higher in Novi Sad than in the Skopje region, suggesting a higher rate of tick exposure in the population of northern Serbia. The difference in seroprevalence may be attributed to climate, geographical landscapes, the prevalence of *Borrelia* spp. infection in questing ticks, and the distribution of tick species in Serbia and North Macedonia.

Although *I. ricinus* is the main tick responsible for human infestations in both countries, *Haemaphysalis inermis* and *Rhipicephalus sanguineus* ticks are more commonly encountered on humans in North Macedonia (authors’ own observation), compared to Serbia [10]. In addition, there have been reports of human infestation by *Hyalomma* ticks in the Skopje region, while no such reports exist for northern Serbia [33]. Similar findings were reported in northeastern Greece, where *Rhipicephalus* spp. and *Hyalomma* spp. ticks are frequently found on humans [34]. Unlike *I. ricinus*, the bites of these ticks will not expose humans to the members of *B. burgdoferi* sensu lato complex and therefore will not induce a humoral response against spirochetal antigens after a blood meal [35], which may impact the overall seroprevalence against specific *Borrelia* spp. antigens.

Although the number of samples processed in our study may not be sufficient for a robust epidemiological assessment of the diseases of interest in North Macedonia, this is the first report suggesting possible exposure to members of the *B. burgdorferi* s.l. complex and/or TBEV in the region. Therefore, we believe that this study is of fundamental importance as a reference for further risk assessment of LB and TBE emergence in North Macedonia and for the surveillance of these diseases in Serbia.

## Figures and Tables

**Table 1:** Distribution of participants according to risk groups.

Risk Group	Patients		Healthy Donors	
	Novi Sad	Skopje	Novi Sad	Skopje
	**Gender**			
Male	23	27	17	12
Female	28	18	45	34
Total	51	45	62	46
	**Age**			
Children	13	5	0	0
Teenagers and adults	32	33	62	46
Seniors	6	7	0	0
Total	51	45	62	46

**Table 2:** Presence of anti-Borrelia IgG and TBEV-neutralizing antibodies in cohorts from Novi Sad and Skopje stratified according to examined risk factors.

	*Borrelia* IgG Seropositive		*Borrelia* IgG Seronegative		TBEV Neutralizing Antibodies Detected		TBEV Neutralizing Antibodies Not Detected	
**Patients Novi Sad**								
	*n*	%	*n*	%	*n*	%	*n*	%
**Gender**								
Male	3	13.04	20	87.96	0	0	23	100
Female	8	28.57	20	71.43	0	0	28	100
**Age groups**								
Children	5	38.46	8	61.54	0	0	13	100
Teenagers and Adults	4	12.5	28	87.5	0	0	32	100
Seniors	2	33.33	4	66.67	0	0	6	100
**Total**	**11**	**21.57**	**40**	**78.43**	**0**	**0**	**51**	**100**
**Patients Skopje**								
	*n*	%	*n*	%	*n*	%	*n*	%
**Gender**								
Male	3	11.11	24	88.89	0	0	27	100
Female	1	5.26	18	94.74	1	5.26	18	94.74
**Age groups**								
Children	0	0	5	100	0	0	5	100
Teenagers and Adults	1	3.03	32	96.97	1	3.03	32	96.97
Seniors	3	42.86	4	57.14	0	0	7	100
**Total**	**4**	**8.89**	**41**	**91.11**	**1**	**2.22**	**44**	**97.78**
**Healthy donors Novi Sad**								
	*n*	%	*n*	%	*n*	%	*n*	%
**Gender**								
Male	2	12.5	14	87.5	0	0	16	100
Female	8	17.4	38	82.6	0	0	46	100
**Age groups**								
Children	0	0	0	0	0	0	0	0
Teenagers and Adults	10	16.13	52	83.87	0	0	62	100
Seniors	0	0	0	0	0	0	0	0
**Total**	**10**	**16.13**	**52**	**83.87**	**0**	**0**	**62**	**100**
**Healthy donors Skopje**								
	*n*	%	*n*	%	*n*	%	*n*	%
**Gender**								
Male	1	8.33	11	91.67	0	0	12	100
Female	0	0	34	100	0	0	34	100
**Age groups**								
Children	0	0	0	0	0	0	0	0
Teenagers and Adults	1	2.17	44	97.77	0	0	46	100
Seniors	0	0	0	0	0	0	0	0
**Total**	**1**	**2.22**	**45**	**97.77**	**0**	**0**	**46**	**100**

## Data Availability

No new data were created or analysed in this study. Data sharing is not applicable to this article.
